# School-based Interventions to Reduce Sedentary Behaviour in Children: A Systematic Review

**DOI:** 10.3934/publichealth.2016.3.520

**Published:** 2016-08-05

**Authors:** Lynda M. Hegarty, Jacqueline L. Mair, Karen Kirby, Elaine Murtagh, Marie H. Murphy

**Affiliations:** 1 School of Sport, Ulster University, Jordanstown, Northern Ireland; 2 Sport and Exercise Sciences Research Institute, Ulster University, Jordanstown, Northern Ireland; 3 School of Psychology, Ulster University, Magee, Londonderry, Northern Ireland; 4 Department of Arts, Education and Physical Education, Mary Immaculate College, University of Limerick, Limerick, Ireland

**Keywords:** sedentary behaviour, intervention, children, school, standing desks

## Abstract

**Introduction:**

Prolonged, uninterrupted periods of sedentary time may be associated with increased risk of Type II diabetes, cardiovascular disease and all-cause mortality even if the minimum recommended levels of daily physical activity are achieved. It is reported that children spend approximately 80% of their day engaged in sedentary behaviours. Since children spend a large portion of their waking time at school, school-based interventions targeting excessive or interrupted periods of sedentary time have been investigated in a number of studies. However, results of the effectiveness of studies to-date have been inconsistent.

**Aim:**

To conduct a systematic review to evaluate the effectiveness of school-based interventions designed to reduce sedentary behaviour on objectively measured sedentary time in children.

**Methods:**

Five electronic databases were searched to retrieve peer-reviewed studies published in English up to and including August 2015. Studies that reported objectively measured sedentary time before and after a school-based intervention to reduce sedentary time were included in the review. Risk of bias was assessed using the Cochrane Collaboration method.

**Results:**

Our search identified eleven papers reporting eight interventions. Studies focused on the physical environment, the curriculum, individual in-class activities, homework activities or a combination of these strategies. Three studies reported decreases in sedentary time following intervention. Study follow-up periods ranged from immediately post-intervention to 12 months. None of the studies were judged to have a low risk of bias.

**Conclusions:**

Multicomponent interventions which also include the use of standing desks may be an effective method for reducing children's sedentary time in a school-based intervention. However, longer term trials are needed to determine the sustained effectiveness of such interventions on children's sedentary time.

## 1. Introduction

“*Sedentary behaviour refers to any waking activity characterised by an energy expenditure < 1.5 metabolic equivalents and a sitting or reclining posture*” [1]. In recent decades, advances in modern technology, increases in passive transportation and shifts in leisure time activities have all contributed to the increasing amount of time both adults and children spend engaged in sedentary behaviours [2],[3].

Uninterrupted sedentary time is increasingly recognised as a distinct health risk behaviour [4]. Spending much of the day sedentary may carry an increased risk to cardiometabolic health even if the minimum recommended levels of physical activity are achieved [5]. In children, sedentary time is positively associated with weight status [6] and obesity [7]. Specific sedentary behaviours such as TV viewing are associated with lower fitness, lower scores of self-esteem and pro-social behaviour, and decreased academic achievement [8].

In the UK children spend approximately 80% of their day sedentary [9] and this behaviour appears to be more prevalent in girls compared with boys [10]. Sedentary time is thought to track from childhood through to adulthood [11],[12] suggesting that sedentary behaviour habits are established at a young age [11]. Colley et al [13] reported that children under the age of 11 years engaged in 1.3 hours less sedentary behaviour than those aged 11–14 years and approximately two hours less than those aged 15–19 years. A systematic review by Jones et al [12] reported moderate-to-large tracking of sedentary behaviour from early childhood up to middle childhood, with a minimum of one-year follow-up from baseline as the inclusion criteria. The tracking coefficients in these studies ranged from 0.35 to 0.60, with a mean of 0.49 and a median of 0.52 [12]. In contrast, Biddle et al [11] reported tracking coefficients ranging from –0.15 (boys over 2 years) to 0.48 (over 1 year) for total sedentary time. In general, children become more sedentary with increasing age [14]. These general increases in sedentary behaviour may be due in part to increased use of computers, smart phones and engagement in social media [15]. In particular, middle childhood and adolescence (9–15 years) has been identified as a life transition or key stage in maturation when parental influence begins to wane and the influence of peers becomes stronger [16]. Interventions aimed at children prior to this milestone, may help prevent further increases in sedentary time.

In addition to sedentary leisure pursuits, school is a key setting for sedentary behaviour [17]. Children spend 57% of their waking time at school [18]. Most primary school children spend approximately 6 hours per day at school [19] with most of this time (65%) being sedentary [20]. School-based interventions have been shown to be effective in reducing health inequalities [21],[22], promoting healthy behaviours generally [23], increasing physical activity [12] and may also be helpful in preventing excessive sedentary behaviour in children [24]. However, there appears to be variability in how each of these studies measured sedentary time as an outcome, therefore leading to uncertainties in objective reliability in intervention efficacy. Nevertheless, the increased availability of portable electronic devices such as accelerometers and inclinometers has enabled the objective measurement of sedentary time [25],[26]. These devices are easy to wear [27] and are more affordable than other objective methods of measurement; for example, direct observation [28] making them more feasible for the evaluation of interventions to reduce sedentary time.

The purpose of this study was to conduct a systematic review of classroom-based interventions designed to reduce objectively measured sedentary time in children.

## 2. Methods

### 2.1. Eligibility Criteria

Studies were assessed for eligibility for inclusion according to the following criteria: (1) reported an intervention aimed at reducing sedentary behaviour which involved the children's classroom; (2) included participants attending primary education; (3) included an objective measure of sedentary time; (4) published in English up to and including August 2015. Interventions which solely or partly aimed to reduce sedentary time were included. Studies which defined sedentary behaviour as “*failure to meet physical activity guidelines*” were excluded. Studies were assessed by one author (LH) with uncertainties referred to a second author.

### 2.2. Search Strategy

Five electronic databases were searched in August 2015 for full-text articles published in peer reviewed journals using a combination of keywords including sedentar*, sitting*, child, child*, teacher, teachers, school and schools. The databases included Medline (OvidSP), PsycINFO, Scopus, CINAHL and Cochrane Libraries. The key search terms included were (1) sedentary behaviour; (2) child; and (3) teacher or school. Table 1 provides the full search strategy which was used in Ovid^®^ Medline which was modified for the remaining databases. The search was also repeated independently by a subject librarian. The reference lists of articles were also searched for suitable articles meeting the criteria.

**Table 1.** Search strategy used in Ovid® Medline.

Searches	
1.	Sedentary Lifestyle/
2.	sedentary.tw.
3.	sitting.tw.
4.	1 or 2 or 3
5.	“teacher*”.tw.
6.	Schools/
7.	(school or schools).mp. (mp = title, abstract, original title, name of substance word, subject heading word, keyword heading word, protocol supplementary concept word, rare disease supplementary concept word, unique identifier)
8.	5 or 6 or 7
9.	exp Child/
10.	(child* or preschool* or pre school*).tw.
11.	9 or 10
12.	4 and 8 and 11

### 2.3. Study Selection

Studies were selected by (1) screening the titles; (2) screening the abstracts; and (3) examining the entire paper if the title and abstract did not provide sufficient information to determine whether it met the inclusion criteria. Studies which did not meet all of the inclusion criteria were discarded.

### 2.4. Changes in Sedentary Time

For inclusion in this review, studies were required, at a minimum, to report a baseline and follow-up objective measurement of sedentary time. These data were required to analyse the effectiveness of the intervention for each study.

### 2.5. Data Extraction and Risk of Bias

Data were extracted in accordance with PRISMA guidelines [29] onto an Excel sheet which was developed for the purposes of this review. This included information on the studies in relation to study characteristics, participant characteristics, intervention characteristics, outcome measurement and data analyses. The Cochrane Collaboration Risk of Bias Tool [30] was used to assess the risk of bias in the studies included in this review across six domains for sequence generation, allocation concealment, blinding, incomplete outcome data and selective reporting. Two authors (LH and EM) independently reviewed the studies and agreed on the risk of bias as low risk of bias, unclear risk of bias or high risk of bias [30]. Discrepancies between the two assessments were resolved by a third author (MM).

### 2.6. Protocol and Registration

The study was registered with PROSPERO, an international database of prospectively registered systematic reviews [31].

## 3. Results

### 3.1. Study Selection

**Figure 1.**
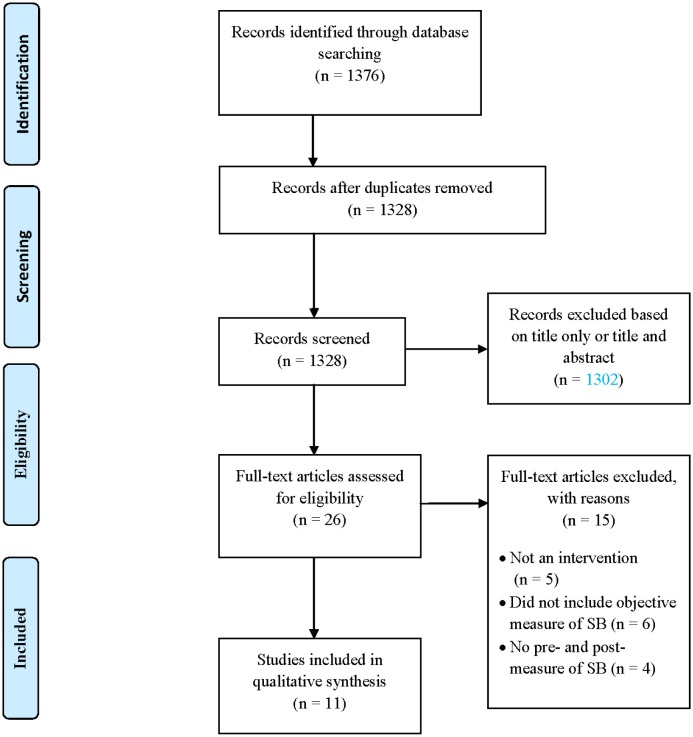
Flow diagram of search results and included studies.

The literature search yielded 1,376 studies. Initially, studies were excluded based on title only, then title and abstract and then full-text using inclusion and exclusion criteria. Articles relating to the same study were grouped together. Eleven papers, reporting eight studies, met inclusion criteria (see Figure 1). The studies included controlled and non-controlled trials.

### 3.2. Study Characteristics

#### 3.2.1. Location

A summary of the included studies are shown in Table 2. Studies originated in the UK (n = 3) [32]–[34], Australia (n = 1) [35],[36], New Zealand (n = 2) [37],[38] and Belgium (n = 1) [39]–[41], whilst one study was based in both the UK and Australia (n = 1) [42]. In seven of the studies, reducing sitting/sedentary time was a main aim [32],[34]–[42], whilst, in another study, reducing sedentary time formed part of an overall school-based intervention to promote health [33].

**Table 2.** Characteristics of included studies.

Studies	Participants				Details of Intervention				
**Authors & Year**	**n**	**Location**	**Age (yrs)**	**Gender**	**Name of Intervention**	**Duration**	**Duration per Session**	**Frequency (days/week)**	**Accelerometer Used**
Aminian et al., (2015) [37]	26	New Zealand	9–11	m f	N/A	9 weeks	Full school day	5	activPAL
Breslin et al. (2012) [32]	416	UK	8–9	m f	Sport for LIFE	12 weeks	1 hour	1	Actigraph
Carson et al. (2013) [35]; Yildirim (2014)[36]	268	Australia	7–9	m f	Transform Us!	18 months	30 mins	5	Actigraph
Clemes et al. (2015) [42]	74	UK and Australia	9–12	m f	N/A	9–10 weeks	UK group: minimum of 1 hour per day. Australian group: full school day	5	activPAL
Fairclough et al. (2013) [33]	318	UK	10–11	m f	CHANGE! Project	20 weeks	60 mins	1	Actigraph
Hinckson et al. (2013) [38]	30	New Zealand	9–11	m f	N/A	4 weeks	Full school day	5	activPAL
Kipping et al. (2014) [34]	2221	UK	8–10	m f	Active for Life Year 5 (AFLY5)	1 school year	Varied	Varied	Actigraph
Verloigne et al. (2012) [39]; Verloigne et al. (2015) [40]; Vik et al. (2015) [41]	372	Belgium^1^	9–11	m f	UP4FUN	6 weeks	Not specified	1–2	Actigraph

^1^ Intervention implemented in Belgium, Germany, Greece, Hungary and Norway but activPAL data related to Belgian sample only.

**Table 3.** Risk of bias in included studies.

	Authors & Year	Adequate sequence generation?	Allocation concealment?	Blinding of participants and personnel?	Blinding of outcome assessors?	Incomplete outcome data addressed?	Free of selective reporting?	Free of other bias?
1	Aminian et al. (2015) [37]	Unclear	Unclear	Unclear	Unclear	Low	Low	High
2	Breslin et al. (2012) [32]	Unclear	Unclear	Unclear	Unclear	Unclear	Unclear	High
3	Clemes et al. (2015) [42]	Unclear	Unclear	Unclear	Unclear	Unclear	Unclear	Unclear
4	Carson et al. (2013) [35]	Low	Unclear	High	Unclear	High	High	Low
5	Fairclough et al. (2013) [33]	Unclear	Unclear	Unclear	High	High	Unclear	Low
6	Hinckson et al. (2015) [38]	Unclear	Unclear	Unclear	Unclear	Unclear	Unclear	High
7	Kipping et al. (2014) [22]	Unclear	Low	Low	Low	Low	Low	Low
8	Verloigne et al. (2012) [39]	Unclear	Unclear	Unclear	Unclear	High	Unclear	High
9	Verloigne et al. (2015) [40]	Unclear	Unclear	Unclear	Unclear	High	Unclear	High
10	Vik et al. (2015) [41]	Unclear	Unclear	Unclear	Unclear	Low	Unclear	High
11	Yildirim et al. (2014) [42]	Low	Unclear	Unclear	Unclear	High	Unclear	Low

#### 3.2.2. Schools

All schools in the studies were state-funded. The socioeconomic status (SES) of the participating schools varied: low SES [32],[37],[38]; a combination of low-, mid- and high SES [34]–[36]; and low and high [33]. One study did not provide the SES of the participating schools [39]–[41]. The study by Clemes et al [42] used a low SES school for the UK group but a mid-high SES school for the Australian group.

#### 3.2.3. Participants

Participants in the eight studies ranged in age from 7–12 years. All studies involved both male and female participants. The number of participants in each study varied. Three studies had less than 100 participants [37],[38],[42]; four studies had 101-500 participants [32],[33],[35],[36],[39]–[41]; and one study had more than 2,000 participants [34]. The samples comprised of multiple ethnicities including white, South Asian, Australian, and Maori.

#### 3.2.4. Sample Size

A convenience sample was used by two studies [37],[38]. One study discussed how sample size was calculated based on a previously published simulation study [35],[36] and a sample size based on intra-cluster correlation coefficients for different outcomes and other information collected during the pilot study [34]. Four studies did not describe how sample size was calculated [32],[33],[39]–[42].

#### 3.2.5. Unit of Allocation and Risk of Bias

The majority of studies used random allocation to intervention or control groups [32]–[36],[39]–[42]. Two studies did not describe how participants were allocated [37],[38]. None of the studies were judged to have a low-risk of bias (Table 3).

#### 3.2.6. Theoretical Basis of the Interventions

Five of the eight studies based the intervention on psychological theory. Social cognitive theory [43] was used by three studies [32]–[34]. One study was developed from the five steps of the Model of Planned Promotion for Population Health, which was based on a socioecological framework [39]–[41]. Another study [35],[36] used a combination of elements of social cognitive theory, behaviour choice theory and the ecological systems theory. Three studies did not describe an underpinning psychological theory in relation to planning the intervention [37],[38],[42].

#### 3.2.7. Duration

The studies included in this review varied in their duration from 4 weeks [38] to 18 months [35],[36]. In one study, it was unclear exactly how many weeks the intervention took place for [34]; however, the corresponding author confirmed that the intervention was spread over a school year [44]. The time of year varied for interventions depending primarily on curriculum demands.

#### 3.2.8. Description of Intervention

Four of the eight included studies consisted of single-component interventions whilst the remainder were multi-component. The multi-component interventions involved parents assisting their children with intervention activities at home [33]–[36],[39]–[41].

Two studies replaced standardised desks and chairs within the classroom with adjustable sit-to-stand desks to reduce sedentary time [37],[38]. Aminian et al [37] also incorporated exercise balls, beanbags and mat space into the classroom. Clemes et al [42] reported two different interventions. In the first study, based in the UK, the teacher rotated the children between standing desks and standard desks whilst, in the Australian-based intervention, all standard desks were replaced with sit-to-stand desks. The remaining five studies involved a combination of specific lessons and activities which focused on reducing sitting time. Sport for LIFE was a healthy lifestyle intervention intended to increase physical activity, decrease sedentary behaviour, reduce screen time behaviours, encourage healthy attitudes and behaviours to nutrition, and reduce body mass index [32]. This intervention involved weekly sessions which consisted of 5–10 minutes of education theory followed by 1 hour of physical activity [32]. The Transform-Us! Intervention aimed to increase physical activity, reduce sedentary behaviour and optimise healthy outcomes [35],[36]. It involved the teacher delivering class lessons which had key learning messages and also included standing lessons, light intensity activity breaks and newsletters. Participants were allocated to one of three intervention groups which focused on (1) reducing sedentary behaviour; (2) increasing physical activity; or (3) reducing sedentary behaviour and increasing physical activity. The CHANGE! Project aimed to assess the effectiveness of the intervention on measures of body size, increasing physical activity, reducing sedentary time and food intake [33]. It consisted of a teacher-led curriculum, learning tasks and homework tasks [33]. The AFLY5 (Active for Life) intervention [34] aimed to increase time spent in moderate or vigorous physical activity, reduce sedentary behaviour, and increase fruit and vegetable consumption. It consisted of weekly lessons and child-parent interactive homework plans. The ENERGY Project aimed to assess the effectiveness of the intervention in reducing children's total sedentary time [39]–[41]. It consisted of 1–2 lessons per week which focused on educating the participants on changing their behaviour in relation to total sitting time [39]–[41]. All studies included a control group that continued with current practice. The interventions were delivered in rural and urban areas.

#### 3.2.9. Delivery of the Intervention

Of the five studies which involved the delivery of specific lessons, four were teacher-delivered lessons and had a specific theme; for example, reducing sedentary time, breaking up sitting time and active transportation [33]–[36],[39]–[41]. One study used specially trained undergraduate and graduate students in partnership with the teachers to deliver the lessons in school [32].

#### 3.2.10. Objective Measure of Sedentary Time

Intervention effects are shown in Table 4. The Actigraph accelerometer was the most commonly used device to measure sedentary time in five of the eight studies [32]–[36],[39]–[41]. The remaining studies used the thigh-worn activPAL device, which quantifies sedentary, upright and ambulatory activities [37],[38],[42].

The majority of the included studies asked the participants to wear the accelerometer for seven consecutive days [32],[33],[37],[38],[42]. Eight consecutive days of wear was required by Carson et al [35],[36] and Verloigne et al [39]–[41]. The shortest wear time was five days by Kipping et al [34]. With the exception of three studies [34],[37],[38], the majority of studies reported compliance issues in relation to adhering to the accelerometer wear time. Consequently, these studies reported valid data for approximately half or less than the number of participants who wore the accelerometer. For data to be deemed valid for statistical analyses, the number of days and the number of hours per day that the accelerometer had to be worn varied between the studies. These included three weekdays and one weekend day [32],[37]; three days [33],[34]; three weekdays [35],[36]; two weekdays and one weekend day [39]–[41]; one weekday [42]; and no minimum wear time described [38]. The minimum number of hours per day included 8 hours [34]–[37],[42]; 9 hours on weekdays and 8 hours on weekend days [33]; 9 hours on weekdays and 11 hours on weekend days [32]; 10 hours on weekdays and 8 hours on weekend days [39]–[41]; and no minimum wear time described [38]. All studies reported time spent sitting in minutes as means and standard deviations.

#### 3.2.11. Pre-, Post- and Follow-up Data

All of the studies included in this review evaluated outcomes pre- and immediately post-intervention but none adopted longitudinal follow-up designs. However, only two studies then collected follow-up data in the months following completion of the intervention. These included 10-weeks post-intervention [33] and 12-months [34]. One other study included a 12-month post-intervention booster [36].

#### 3.2.12. Use of Incentives

The studies included in this review either did not offer, or did not disclose the use of incentives to reward the participants for taking part. However, as part of the methodologies, participants in some studies did receive resources. For example, to compensate for the standing desks having no drawers, the participants were provided with shoulder bags [37]. One study suggested that incentives should be used in future studies especially to increase adherence to the wearing of accelerometers [32].

#### 3.2.13. Sedentary time

Five of the eight studies reported decreases in sedentary time between baseline and post-intervention measurements [32],[35],[37],[38],[42]. Aminian et al [37] reported that, on weekdays, during waking hours, there was an overall decrease in sitting time by 45 minutes from baseline (9.56 ± 1.27 hours) to post-intervention (7.64 ± 2.06 hours) and an increase in standing time by 55 minutes in the experimental group from baseline (3.71 ± 0.92 hours) to post-intervention (3.71 ± 0.92 hours). During school hours, sitting time reduced by 36 minutes in the experimental group (3.88 ± 0.36 hours at baseline compared to 2.81 ± 0.36 hours post-intervention) [37]. However, the authors noted wide confidence limits and that the results were unclear. Further decreases in sitting time were observed during other segments of the day between pre- and post-intervention in the experimental group including before school (0.86 ± 0.42 v. 0.59 ± 0.37 hours per day) and after school (4.82 ± 1.15 v. 4.15 ± 1.67 hours per day).

Breslin et al [32] reported an overall decrease in sedentary time for the intervention group of approximately 25.5 minutes per day (709.28 ± 41.32 minutes per day at baseline and 684.30 ± 72.17 minutes per day post-intervention). However, further analyses of sedentary time in the intervention group at specific intervals showed varying results. Sedentary time increased before school (+0.71 minutes per day), during school (+9.59 minutes per day) and after school whilst a decrease was observed in the time period between 6pm and bedtime (–40.24 minutes per day) [32]. Further analysis by Breslin et al [42] showed a significant multivariate effect for sedentary behaviour for the intervention group (F(4,56) = 14.416; *p* < 0.001; *ηp*^2^ = 0.507). During particular segments of the day, the participants showed statistically significant differences for average time in sedentary behaviour at follow-up compared with baseline. Specifically, between 15:00 and 18:00 (F(1,59) = 4.906; *p* < 0.031; *ηp*^2^ = 0.077) which represented an increase in sedentary behaviour and between 18:00 and bedtime (F(1,59)=38.821; *p* < 0.001; *ηp*^2^ = 0.397) which represented a decrease in sedentary behaviour.

The mid-intervention results by Carson et al [35] at 5–9 months reported a statistically significant decrease of 13.3 minutes per day in weekday sedentary time in the arm of the intervention group which focused on reducing sedentary time and increasing physical activity in comparison to the control group. However, there were no statistically significant decreases observed during class time. The total effect on total weekday sedentary time was –13.28 (–24.37, 2.20) (95% CI) [35].

The results for the intervention by Clemes et al [42] reported that the proportion of time spent sitting in class decreased significantly at follow-up in both intervention groups. In the UK study which had some standing desks, classroom sitting time decreased by –52.4 ± 66.6 minutes per day in the intervention group and –6.9 ± 91 minutes per day in the control group [42]. The Australian study with all standing desks showed a decrease of –43.7 ± 29.9 minutes per day in the intervention group and –28.2 ± 28.3 in the control group [42]. Sitting time as a percentage of wear time decreased in both intervention groups by –9.8 ± 16.5% (UK sample) and –9.4 ± 10% (Australian sample). The results for both intervention groups showed statistical significance. Overall decreases in sitting time were also reported for both intervention groups (UK: –80.8 ± 103.4 minutes per day; Australia: –68.3 ± 97.2 minutes per day); however, these results were not statistically significant.

Fairclough et al [33] reported that participants recorded less sedentary time at follow-up; however, post-intervention, they did over 28 minutes more per day but these results were not statistically significant. Baseline sedentary time was also reported for the comparison and intervention groups but not post-intervention. Instead, multi-level analyses of the effectiveness of the intervention between baseline and post-intervention were conducted. Adjusted analyses showed no significant intervention between-group intervention effects for sedentary time.

Results by Hinckson et al [38] showed a decrease in sitting time of approximately 60 minutes (9.26 ± 1.15 hours per day in the experimental group at baseline compared to 8.27 ± 1.45 hours per day post-intervention). Mean sitting time decreased by 0.3 hours in the control group and by 0.99 hours in the experimental group. Hinckson et al (2013) reported this difference to have an effect size of –0.49 which was a likely small decrease.

Kipping et al [34] reported the difference in means as –0.11 (–9.71 to 9.49) minutes per day for sedentary time when comparing the intervention and control group but this was not statistically significant. Although the remaining UP4FUN study by Verloigne et al [39]–[41] reported no significant differences with regards to changes in sedentary time, it did observe increases in sedentary time which were statistically significant; the percentage of time spent sedentary from pre- to post-intervention in the intervention group for total time (63.3% v. 66.5%), weekday (64.1% v. 66.7%), weekend day (62.2% v. 66.4%); and after school (60.8% v. 65.8%) [39]. However, no statistically significant differences were reported for during school hours (62.4% v. 62.9%). Table 4 provides a summary of the intervention effects of the included summaries. Only two studies aimed to interrupt sitting time at regular intervals throughout the day [35],[39].

The different findings of the included studies are likely to be attributable to variations in (1) the intervention strategies used; (2) the duration and frequency of the interventions; (3) the follow-up period; (4) the sample size; (5) the theoretical models used to plan the interventions; (6) minimum wear time required for data to be considered valid; (7) the definition of non-wear time; (8) the type of monitor used; and (9) how data were cleaned.

The intervention strategies included in this review ranged from single-component to multi-component interventions and it is unknown which is more effective for reducing sedentary time in children. This is mirrored in relation to increasing total daily physical activity as there is currently limited evidence on the effectiveness of multicomponent interventions [45]. In relation to obesity, which is a multi-factorial problem, approaches must target individual and environmental factors that promote healthy behaviours [46]. However, single-component studies have also been shown to positively affect adiposity outcomes in children [47].

The duration of studies included in this review ranged from 4 weeks to 18 months. Verloigne et al [39] discussed this issue suggesting that a shorter intervention period may make the intervention more feasible; however, they also refer to a meta-analysis by Biddle et al. [48] which concluded that interventions to reduce sedentary time of less than 4 months' duration showed small treatment effects. The frequency of the interventions included in this review ranged from 1 hour per week to daily exposure and, because the literature has not adequately ascertained the dose-response effect of the number or duration of breaks in sedentary time [49], it is difficult to clearly provide guidelines on these aspects of future interventions.

A meta-analysis by Biddle et al [48] has highlighted the variation in follow-up periods in sedentary behaviour interventions in young people given that only five out of seventeen included studies had a follow-up assessment and those that did had a short follow-up period. Therefore, the meta-analysis recommended longer follow-up with large samples.

It is clear from this review that a huge variation in sample size existed in the included studies. Given the potential for too small or too large sample sizes to affect the power of the study, it is possible that some of the included studies may have been under-powered to clearly detect between-group differences of statistical significance [50],[51].

Social cognitive theory [43] was the most commonly referred to model used to plan the interventions. However, some of the included studies which reported statistically significant reductions in sedentary time did not describe a model used at the planning stage of their interventions.

Minimum wear-time varied in the included studies in terms of the number of days that accelerometers had to be worn ranging from one day to four days, weekdays only to weekday and weekend days. Also, the minimum number of hours for which the data was deemed valid ranged from 8 hours to 10 hours.

Given the variation in the definitions of non-wear time used in the included studies, it is possible that sedentary time may have been under- or over-reported — an issue highlighted by Janssen et al [52]. Actigraph was the most commonly used accelerometer used in the included studies. Although it has a built-in inclinometer, the Actigraph cannot clearly distinguish between standing and sitting and, therefore, it is possible that sedentary time may have been under- or over-reported [53]. All included studies did not report specifically how data were cleaned and reduced; therefore, the potential exists for results to vary within studies depending on the methodology used.

**Table 4.** Intervention effects of the included studies.

Authors & Year	Nature of Intervention	Intervention Effects
Aminian et al. (2015) [37]	Standing workstations replaced traditional desks. Exercise balls, beanbags and mat space.	Overall mean sitting time (hours; mean ± SD): Intervention: pre, 9.56 ± 1.27; post, 7.64 ± 2.06. Control: pre, 9.34 ± 1.32; post, 8.08 ± 3.10. Mean sitting time during school (hours; mean ± SD): Intervention: pre, 3.88 ±0.36; post, 2.81 ± 0.36. Control: pre, 3.59 ± 0.45; post, 3.24 ± 0.81. Decrease in weekday sitting time: 45 minutes (CL ± 122). Moderate reduction by possibly 36 minutes in sitting time during school but results unclear.
Breslin et al. (2012) [32]	Education theory and activities led by teacher.	Intervention group: lower levels of sedentary behaviour at follow-up compared to baseline. Significant main effects: F(1,49) = 5.585; *p* < 0.022; *ηp*^2^ = 0.102. Increase in sedentary behaviour 15:00–18:00. Decrease in sedentary behaviour 18:00–bedtime. Significant multivariate effects: Intervention group, sedentary behaviour: F(4,56) = 14.416; *p* < 0.001; *ηp*^2^ =0.507. Time period: 15:00-18:00, F(1,59) = 4.906; *p* < 0.031, *ηp*^2^ = 0.077 and 18:00-bedtime, F(1,59) = 38.821; *p* < 0.001; *ηp*^2^ = 0.397).
Carson et al. (2013)[35]; Yildirim (2014)[36]	Combination of strategies using the curriculum, in-house activities, physical environment & home setting	13.3 minute decrease in weekday sitting time in arm of intervention that aimed to increase PA and reduce SB (significant). Total effect on total weekday sedentary time was –13.28 (–24.37, 2.20) (95% CI).
Clemes et al. (2015) [42]	UK study: participants were rotated around 6 sit-to-stand desks so that they stood for at least one hour per day. Australian study: sit-to-stand desks replaced all traditional desks and participants stood for a minimum of 30 mins per day. In both studies, teachers provided information on benefits of reducing sedentary behaviour and classroom sitting time.	Sitting during class (% of wear time): UK sample: Intervention: pre, 71.8 ± 10.6; post, 62 ± 15.8 (*p* = 0.03). Control: pre, 68.6 ± 20; post, 65.4 ± 20.1 (not significant). Australian sample: Intervention: pre, 67.9 ± 8.4; post, 58.5 ± 8.4 (*p* < 0.001). Control: pre, 70.8 ± 5.8; post, 64.8 ± 10.8 (*p* = 0.04).Weekday sitting time (minutes): UK sample (mean ±SD): Intervention: pre, 606.5 ± 66.4; post, 525.7 ± 103.7. Control: pre, 566.1 ± 92.6; post, 574 ± 180.6. Australian sample (mean ±SD): Intervention: pre, 498.2 ± 80.2; post, 429.8 ± 60.4. Control: pre, 489.7 ± 84.6; post, 435.5 ± 81.2.
Fairclough et al. (2013) [33]	Teacher-led curriculum, learning resources, homework tasks, CD-ROM. Teacher received 4 hours of training in the delivery of the curriculum resource.	Compared with baseline data, 28 minutes more sedentary time in intervention group post-intervention but 8.5 minutes less at follow-up. Effect for sedentary time: *β* = –8.44 (95% CI = –53.23, 36.35) minutes (non-significant).
Hinckson et al. (2015) [38]	Standing workstations replaced traditional desks.	Sitting Time (hours; mean ± SD): Intervention: pre, 9.26 ± 1.15; post, 8.27 ± 1.45. Control: pre, 9.30 ± 1.46; post, 9.00 ± 0.80. Effect size (90% CL): 0.49 (0.64).
Kipping et al. (2014) [34]	Teachers trained to deliver 16 sessions — 10 of which had associated homework. Child-parent interactive homework tasks also.	Difference in means between intervention and control group of -0.11 (–9.71 to 9.49) minutes per day less sitting (non-significant).
Verloigne et al. (2012) [39]; Verloigne et al. (2015) [40]; Vik et al. (2015) [41]	Teacher delivered 1–2 intervention lessons per week. Manual given to teachers who were trained by researcher. Each week had a specific theme.	Effects: Sedentary time (*β* = 2.18, SE = 0.59, d = 0.20); weekday sedentary time (*β* = 1.07, SE = 0.47, d = 0.12) (significant); weekend day sedentary time (*β* = 4.17, SE = 0.88, d = 0.25) (significant); school hours (*β* = 0.22, SE = 0.46) (non-significant); after school sedentary time (*β* = 3.32, SE = 0.67, d = 0.26) (significant).

In four of the five studies which used the Actigraph accelerometer, sedentary behaviour was defined as less than 100 counts per minute [33]–[36],[39]–[41]. Non-wear time would have been applicable to all eight studies. However, not all studies discussed this aspect of their statistical analysis. Four studies defined non-wear time as 20 minutes or more of consecutive zeros [32],[33],[35],[36],[39]–[41],[54]. Two studies used a definition of 60 minutes or more of consecutive zeros [34],[42]. Aminian et al [37] provided participants with a log to record when the activPALs were removed. When the data were analysed, if there were non-wear times which did not correspond with the participant's log, the non-wear times were classified as missing and the data were then excluded from the analyses. One study did not report on non-wear time [38].

## 4. Discussion

### 4.1. Summary of Evidence

This is the first systematic review to examine evidence on the effectiveness of sedentary behaviour interventions based in the classroom to reduce objectively measured sedentary time in children. Three of the eight studies included in this review observed differences in sedentary time [32],[35],[42]. These decreases were also observed in ethnically diverse and also low- [32],[35],[42], middle-high [42] and high-SES [35] groups. It should be noted that the largest decrease reported was in a study which mainly focused on replacing all classroom standardised desks and chairs with sit-to-stand desks [42].

Reasons for lack of effect on sedentary time in some of the remaining interventions may have included the time lapse between the feasibility study and intervention [34]; the lack of emphasis on educational messages on sedentary behaviour [33]; and the use of accelerometers which could not differentiate between sitting and standing [39]. Verloigne et al [39] hypothesised that sedentary time could have been replaced by low-intensity physical activity (LPA); however, no significant effect was found in their study whereas Fairclough et al [33] reported a statistically significant increase in LPA at follow-up. Kipping et al [34] reported the lowest decrease in overall sitting time; the difference in means between the intervention and control groups were –0.11 (–9.91 to 9.49) minutes per day. Clemes et al [42] reported the largest decrease in overall sitting time (80.8 ± 103.4 minutes per day and 68.3 ± 97.2 minutes per day) followed by 60 minutes less per day in the study by Hinckson et al [38] and 45 minutes less per day in the study by Aminian et al [37]. However, these results were not statistically significant. Only two of the included studies aimed to interrupt sitting time [35],[39]. Given that uninterrupted sedentary time is increasingly recognised as the distinct health risk behaviour as opposed to just the total time spent sedentary [4], future studies examining the effects of interventions in interrupting sedentary time are warranted.

Although the evidence suggests that multi-component interventions using a combination of a teacher-led curriculum, specialist lessons, homework tasks (with or without the assistance of parents) and printed materials may be effective in reducing sedentary time in children, the inclusion of standing desks may be more effective. The evidence presented suggests that the approach of using standing desks removes the conscious effort of the users to engage in a healthy behaviour (standing) and decrease the negative behaviour (prolonged sitting). Therefore, the studies which used the standing desks consequently removed a competing alternative behaviour from the equation (i.e. sitting) by not having traditional desks available. This is supported by Koepp et al [55] who reported that standing desks may provide an attractive alternative to traditional seated desks as there is less potential for sitting. However, it should be noted that the standing desks used in the studies included sit-to-stand desks which could be adjusted for sitting or standing. These results may be relevant to policymakers, school management teams, teachers and health promotion departments.

It is evident from the included studies that a child's physical environment, such as the school setting, can act as an important catalyst towards reducing sedentary time. The British Heart Foundation [56] has emphasised the need for policy makers to encourage schools to reduce extended periods of sitting for pupils. Of all the strategies included in this review, the use of adjustable standing desks produced the largest decrease in sitting time [37],[38],[42]. The World Health Organization (WHO) [57] states that interventions that are based on theory are more likely to be effective. Although the aforementioned studies did not explicitly discuss a theoretical model, it is proposed here that the Social Ecological Model [58], which focuses on the importance of the child's environment (individual, physical, social and policy) could be applied to not only assist our understanding of these findings, but may assist in designing future interventions. This is important because, within the social ecological framework, a combination of individual, environmental and policy interventions are required to achieve sustainable and substantial positive health behaviour change [59]. A child's family also falls under the ecological theory which would explain why a number of the interventions in the included studies also involved, for example, homework tasks for the children to complete with their parents. However, future research may want to explore the effectiveness of using parents to reduce the sedentary time of their children. If a child's environment within the classroom can be structured to facilitate less sitting and more standing, then consideration must be given as to how the traditional classroom may be structured away from the use of traditional desks and chairs. This is important for policymakers, schools and teachers as each can play a role in mitigating the effects of prolonged sitting. However, notably, none of the included studies examined the economic costs of the interventions which is vital for policymakers.

### 4.2. Risk of Bias

Although the majority of studies used random allocation to intervention or control groups, most of them lacked detail as to how this was achieved. This could possibly have been a confounding variable in the studies. However, given the nature of the studies, it would be impossible to blind participants to intervention and control conditions. Having distinct arms of varying exposure may help reduce bias in future research. Randomisation may not always be possible in school-based clustered randomised trials [60]. However, these studies are still at a high risk of bias because participants in the intervention and the control groups would have been aware which condition they were assigned to. Concealment can be achieved, for example, by using sequentially numbered, opaque, sealed envelopes. Waters et al. [60] describes studies to be at low risk of bias if the investigators enrolling participants are blinded to group assignment. A recent Cochrane Review [61] investigating the effect of interventions to reduce sitting time at work did not assess blinding of participants or personnel for risk of bias as it is not possible to blind either in studies that are trying to modify activity behaviour. Shrestha et al [61] also judged studies to be at high risk of bias if they did not report concealing intervention versus control group allocation.

### 4.3. Strengths and Limitations

The main strength of this systematic review was that only studies which objectively measured sedentary time were included in comparison to less objective measures, for example, child- or parent-report, which are subject to either over- or under-reporting [62]. A limitation of this review is that the studies which were included were assessed by one author increasing the possibility that a study has been missed.

## 5. Conclusions

The evidence suggests that a multi-component intervention which includes the use of standing desks may have a small effect in reducing sedentary time in the school-setting. However, these conclusions are advanced on the basis of a small number of studies. There is no evidence currently on the longitudinal effects of sedentary behaviour interventions in a school setting and more research is needed on the long-term effectiveness of school-based interventions to reduce and interrupt sitting time in children. Furthermore, given the variation in the duration of included interventions, future research should examine the optimal length required to be effective. Considering the need to form healthy habits such as reducing sitting time, exposure on a daily basis may be preferred for use in an intervention compared to once weekly. Accelerometers which can clearly differentiate between time spent sitting/lying, standing and stepping should be the preferred choice for objectively measuring sedentary time. Also, consistent/strict criteria as to what constitutes valid accelerometer data should be developed and how participants can be encouraged to meet minimum wear criteria should be examined. Researchers should also provide a clear account as to how accelerometer data are filtered and cleaned. A standard definition of non-wear time which is appropriate to the age of the participants will help achieve consistency when comparing results across studies. Collectively, these recommendations may be achieved through more multi-component, cluster randomised controlled trials which consider these methodological aspects including adequate sample size with a power analysis which involve parents and school to reduce sedentary time in children.
